# Oxyresveratrol Improves Cognitive Impairments and Episodic-like Memory through Modulating Neuroinflammation and PI3K-Akt Signaling Pathway in LPS-Induced Mice

**DOI:** 10.3390/molecules29061272

**Published:** 2024-03-13

**Authors:** Guangling Yin, Chunxing Pan, Hong Liu, Changzhi Dong, Xia Chang, Wei Zhou, Shanshan Wang, Zhiyun Du

**Affiliations:** 1 School of Biomedical and Pharmaceutical Sciences, Guangdong University of Technology, Guangzhou 510006, Chinachunxing0221@163.com (C.P.);; 2 Byhealth Co., Ltd., Guangzhou 510030, China; 3 Jihua Laboratory, Foshan 528061, China; 4 Foshan Allan Conney Biotechnology Co., Ltd., Foshan 523281, China

**Keywords:** oxyresveratrol, cognitive impairments, memory, polarization, PI3K-Akt

## Abstract

Oxyresveratrol is one of the active ingredients derived from mulberry branch with strong anti-inflammatory bioactivity. In this research, we want to explore if oxyresveratrol can improve cognitive impairments and episodic-like memory and its mechanism. In LPS-induced BV-2 cells, 25 μM OXY can significantly inhibit the expression of NO and alter the M1/M2 polarization by regulating M1/M2 phenotype makers. In vivo, OXY (50, 100 mg/kg) significantly reversed cognitive impairments and alleviated neuronal injuries caused by neuroinflammation. According to network pharmacology analysis, OXY alleviated neuroinflammation via the PI3K-Akt pathway. In general, the research revealed that OXY can improve cognitive impairments and episodic-like memory through alleviating LPS-induced neuroinflammation and regulating the PI3K-Akt signaling pathway.

## 1. Introduction

Neuroinflammation refers to the immune response that occurs in the central nervous system (CNS) of the brain, which is involved in immune response by regulating microglia, astrocytes, and CNS neurons. It is extensively involved in most of neurodegenerative disorders, including Alzheimer’s disease, Parkinson’s disease, multiple sclerosis, and others [1], accompanied by symptoms of cognitive impairment and memory loss. Various stimuli include amyloid β (Aβ), tau protein, and other neurotoxic metabolites are crucial igniters of neuroinflammation [2].

Microglia constitute 5–12% of CNS cells, depending on the region. They are the principal resident immune cells of the brain and are involved in homeostasis and in host defense against pathogens and CNS disorders [3]. During postnatal development, microglia eliminate redundant neurons that do not establish functional circuits. Importantly, microglia shape neuronal synapses by phagocytosing dendritic spines that are not receiving inputs from synaptic contacts [4,5]. Microglia also mediate synaptic pruning via CX3CR1, which interacts with CX3CL1, a transmembrane glycoprotein expressed on the neuronal surface that is also released as a soluble molecule after proteolytic cleavage. Lack of CX3CR1-CX3CL1 interactions curtails the engulfment of PSD95-immunoreactive postsynaptic densities and ultimately impairs connectivity and afferent synaptic inputs in the mouse hippocampus [6]. Once the microglia are activated, they will differentiate into two phenotypes, M1 and M2. The microglia of M1 phenotype releases inflammatory cytokines, such as interleukin-1β (IL-1β), IL-6, and tumor necrosis factor alpha (TNF-α) to activate astrocytes and other microglia, resulting in inflammation amplification. These neurotoxic factors may directly lead to neuronal injuries or death. On the contrary, M2 phenotype microglia produces anti-inflammatory cytokines and mediators which are neuroprotective. Targeting the imbalance of M1/M2 microglia polarization has been suggested as an emerging therapeutic strategy to combat neuroinflammation-related diseases [7].

Microglia responds immediately to any changes to defense against pathogenic invasion and infection in the CNS [8,9,10]. The PI3K family is a lipid kinase, Akt (also known as protein kinase B, PKB) is from the AGC kinase family, and mTOR is a serine-threonine kinase. Either mTORC2 or PDK1 can activate Akt phosphorylation. The phosphorylation of Akt is accompanied by the phosphorylation of mTOR, which in turn activates the phosphorylation and degradation of the downstream signaling NF-κB inhibitory protein IκB kinase, and at last leads to the activation of NF-κB and promotes the resveratrol expression of inflammatory molecules iNOS and COX-2 [11].

**Figure 1:**
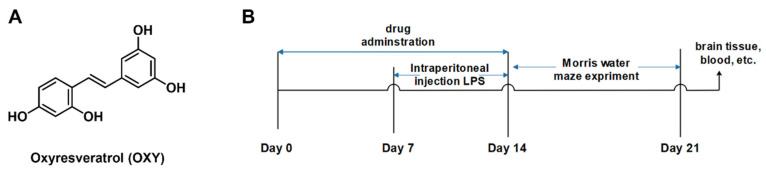
(**A**) Chemical structure of oxyresveratrol (OXY) and (**B**) experimental flowchart of the neuroinflammation model mice.

A mounting number of studies have reported molecules from natural resources [12,13] or organic synthesis [14,15] that are capable of inhibiting neuroinflammation. Oxyresveratrol (Figure 1A, OXY, PubChem CID:5281717), which is widely found in mulberry, jackfruit, myrtle, smilax, vine plants and veratrol, is a stilbene polyphenol [16,17]

OXY is structurally similar to resveratrol (PubChem CID: 445154) and is a hydroxylated derivative of resveratrol at the 2′ position. Of note, the biological activities of OXY are similar to resveratrol, such as anti-inflammatory, antioxidant, antitumor and neuroprotective activities. Resveratrol has been extensively explored as an anti-neuroinflammatory agent. However, the neuroinflammatory properties of OXY have not yet been fully characterized. Researchers have found that OXY has better water solubility, faster oral absorption rate, and longer metabolism time than resveratrol [18].

In this study, we established neuroinflammation models in vitro and cognitive and memory impairment model in vivo. The anti-neuroinflammatory effect of OXY was explored. Network pharmacology analysis and signaling pathway verification were introduced to rationalize the mechanism-of-action of OXY.

## 2. Results

### 2.1. Effect of OXY on the Production of NO and Polarization in BV-2 Cells

**Figure 2:**
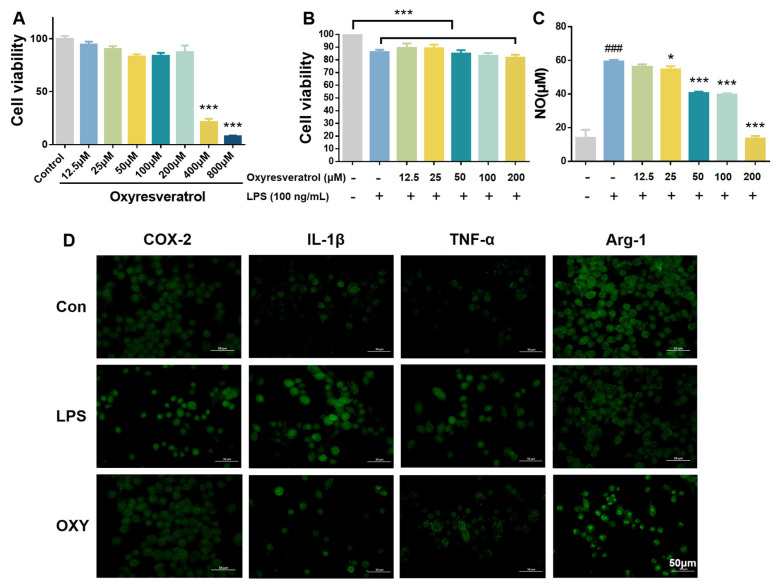
OXY inhibited NO release and altered the M1/M2 polarization markers on BV−2 microglia. (**A**,**B**) The cytotoxicity of OXY on BV−2 cells. (**C**) The inhibitory effect of OXY on NO production. (**D**) OXY modulated phenotype polarization markers on BV−2 cells (M1: IL−1β and TNF−α; M2: Arg−1). All values are mean ± S.D. (*n* = 4). * *p* < 0.05, *** *p* < 0.001 versus model group, ^###^ *p* < 0.001 versus control group. One−way ANOVA followed by Tukey’ s comparison test.

Microglial cells serve as the predominant parenchymal immune cells of the brain. The potential cytotoxicity of OXY on BV-2 microglia cells was first investigated using MTT assay. As shown in Figure 2A, OXY showed little cytotoxicity on BV-2 cells when the concentration was below 200 μM. Then, BV-2 cells were co-cultured with LPS and OXY. As shown in Figure 2B, the slight decrease in cell viability was observed when stimulated with LPS, and it could be partially relieved by OXY.

In neuroinflammation, inducible nitric oxide synthase (iNOS) catalyzes the conversion of *L*-arginine to nitric oxide (NO). In this study, BV-2 cells were stimulated by LPS to establish a model of microglia activation in vitro, which showed enhanced expression of iNOS and production of NO. Then, the concentration of NO by BV-2 cells was determined by Griess Reagent. As shown in Figure 2C, after LPS treatment (100 ng/mL) for 24 h, the content of NO in BV-2 cell culture medium increased significantly. OXY inhibited the production of NO on BV-2 cells in a dose-dependent manner. At the concentration of 50 μM, compared with the model group, OXY showed an inhibition rate higher than 50%.

Then, inflammation-related markers were measured on LPS-stimulated BV-2 microglia by immunofluorescence to explore the phenotype of microglia polarization. As shown in Figure 2D, the production of pro-inflammatory phenotype markers, IL-1β and TNF-α, which represented the M1 polarization of microglia, were greatly increased after LPS stimulation while the M2 anti-inflammatory phenotype marker (Arg-1) was decreased. Compared with the model group, OXY treatment significantly suppressed the release of M1 pro-inflammatory cytokines (IL-1β and TNF-α), and increased the production of Arg-1, the M2 anti-inflammatory marker.

### 2.2. OXY Improved Behavioral Impairments in LPS-Induced Mice

**Figure 3:**
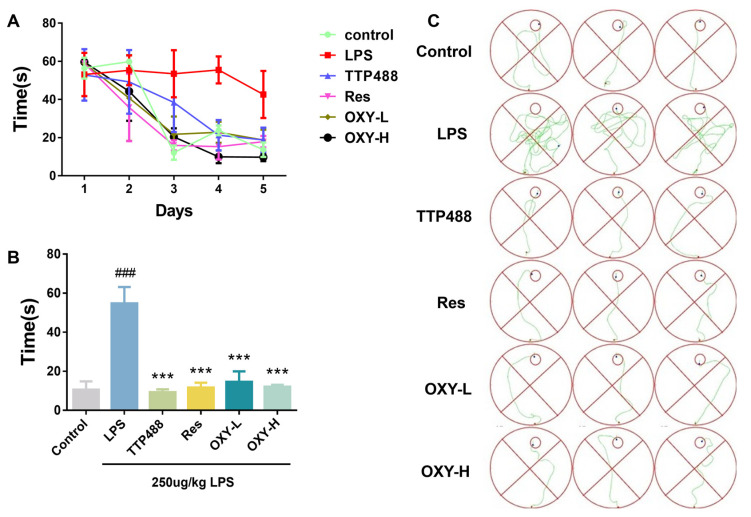
OXY improved cognitive impairments and behavioral deficits in LPS−induced mice (directional navigation experiment). (**A**) The time for finding the survival platform in training session. (**B**) The time for finding the survival platform in directional navigation experiment. (**C**) Trajectories of directional navigation. All values are mean ± S.D. (*n* = 6), *** *p* < 0.001 versus LPS group, ^###^ *p* < 0.001 versus control group. One-way ANOVA followed by Tukey’ s comparison test.

Continuous exposure to LPS via intraperitoneal injection for 7 days was assumed to provoke neuroinflammation and cause cognitive impairments and behavioral deficits in mice [19]. The effect of LPS on neurobehavioral ability was assessed by the Morris water maze experiment. In the directional navigation experiment, as shown in Figure 3A,B, the LPS group took a much longer time to find an underwater survival platform compared with the control group. Meanwhile, the trajectory of directional navigation was significantly more complex in the LPS group than in control group (Figure 3C), which meant that the intraperitoneal injection of LPS induced cognitive impairment and behavioral deficits in the model. However, administering 5 mg/kg TTP488, an anti-neuroinflammatory RAGE inhibitor in clinical trials for Alzheimer’s disease, significantly shortened the swimming time and distance to locate the platform. As shown in Figure 3, like TTP488 and Res, consecutive administration of OXY for 14 days also reduced the time to find the platform in both the training session and the formal experiment, and the navigation trajectories of mice in OXY group were significantly more direct than that of the LPS group.

**Figure 4:**
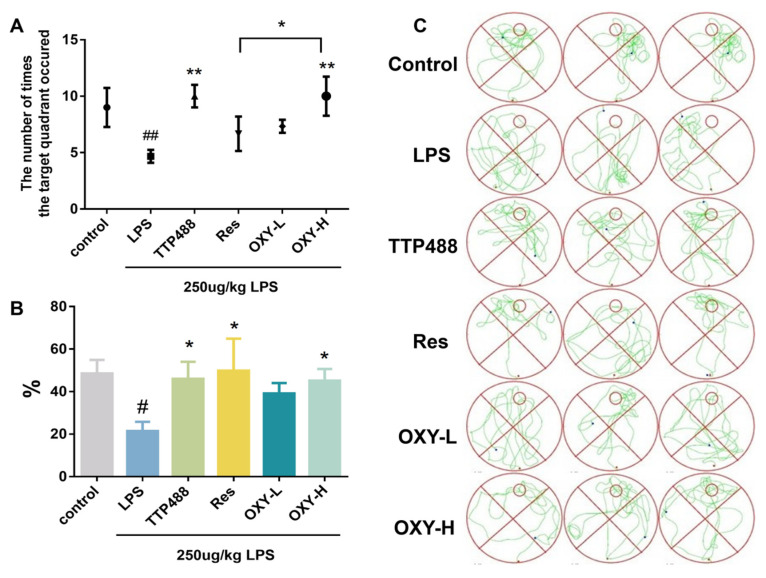
OXY cognitive impairments and behavioral deficits in LPS−induced mice (space exploration experiment). (**A**) The frequency of crossing the target quadrant. (**B**) The ratio of swimming time spent in the target quadrant. (**C**) The trajectories of space exploration. All values are mean ± S.D. (*n* = 6), * *p* < 0.05, ** *p* < 0.01 versus LPS group, ^#^ *p* < 0.05, ^##^ *p* < 0.01 versus control group. One-way ANOVA followed by Tukey’ s comparison test.

In the space exploration experiment, the survival platform was removed, and the experimental mice would spontaneously search for the missing survival platform. Then, the time spent in the target quadrant and the frequency of crossing the target quadrant were recorded. As shown in Figure 4A,B, the time spent in the target quadrant and the frequency of crossing the target quadrant among the mice in LPS group were significantly lower than for mice in the control group. Conversely, mice in the OXY group, Res group, and TTP488 group, remarkedly relieved the decreases in spontaneous activities in LPS−induced mice. According to the results of the Morris water maze experiments, OXY treatment significantly improved cognitive impairments and behavioral deficits in neuroinflammation model mice.

### 2.3. OXY Alleviated LPS-Induced Nerve Cells Injury

**Figure 5:**
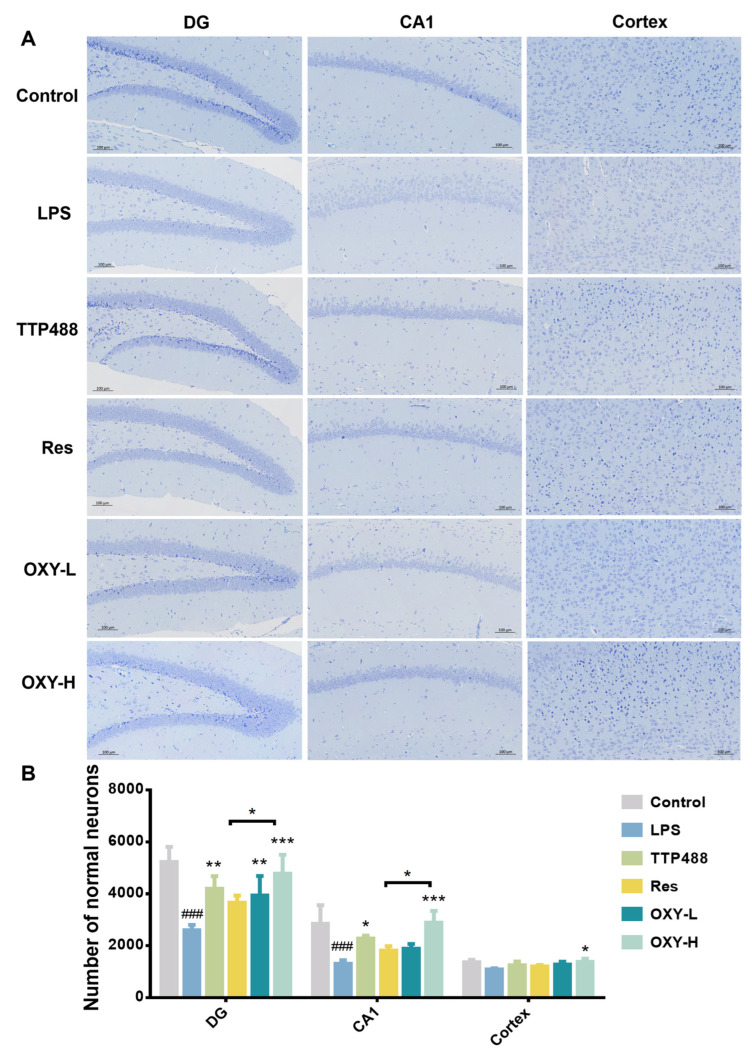
(**A**) Nissl staining of DG and the CA1 part of hippocampus and cortex. (**B**) Quantitative analysis. All values are mean ± S.D. (*n* = 3), * *p* < 0.05, ** *p* < 0.01, ***p < 0.001, treatment groups versus LPS group, ^###^ *p* < 0.001, LPS group versus control group. One-way ANOVA followed by Tukey’ s comparison test.

To explore the protection activity of OXY against neuronal injury by neuroinflammation in the hippocampus of mice, the Nissl staining of the DG part and CA1 part of the hippocampus and cortex were analyzed. As shown in Figure 5, a large number of Nissl bodies were distributed in the DG area, CA1 area and cortex area of the hippocampus of mice in the control group, and the slices showed a darker blue. The nuclei of neurons were clearly visible, and the neurons were closely arranged in the CA1 area. Compared with the control group, the number of Nissl bodies distributed in the hippocampus and cortex of the mice in the LPS group was significantly lower than that of the control group, and the slices showed a lighter blue. It could also be noticed that the cell membrane is incomplete, the nucleus was blurred, and the arrangement of neurons in the CA1 area is sparse and scattered. However, after the administration of resveratrol and OXY, the number of Nissl bodies in neurons increased significantly. Using Image J (1.4.3.67) software to quantitatively analyze the number of normal neurons, the results showed that the neurons in the DG area and CA1 area of the hippocampus of the LPS group were largely apoptotic compared with the control group. After treatment with OXY, resveratrol or TTP488, the number of alive neurons was increased considerably. Compared with the same dose of resveratrol, OXY showed a superior therapeutic effect.

### 2.4. OXY Alleviated LPS-Induced Oxidative Stress in Brain

**Figure 6:**
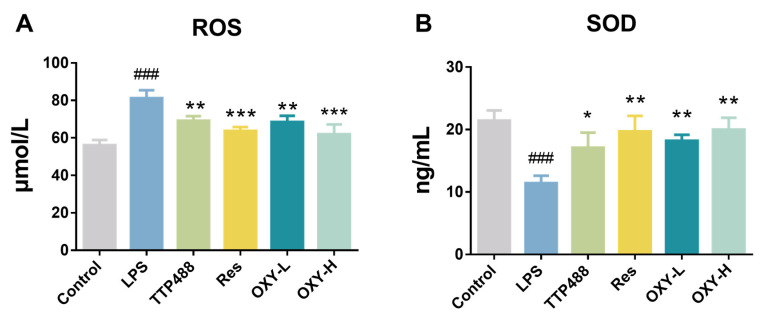
The expression levels of (**A**) ROS and (**B**) SOD in the mice brain. All values are mean ± S.D. (*n* = 3), * *p* < 0.05, ** *p* < 0.01, *** *p* < 0.001, treatment groups versus LPS group, ^###^ *p* < 0.001, LPS group versus control group. One-way ANOVA followed by Tukey’ s comparison test.

In the study, OXY was also found to regulate LPS−induced oxidative stress. As shown in Figure 6A, compared with the control group, the expression of ROS in the LPS group was significantly higher. After treating with OXY, the expression of ROS was reduced significantly in a dose–response relationship. As shown in Figure 6B, OXY was also found to alleviate the reduction of the SOD expression by the LPS stimulation. These findings suggest that OXY could inhibit LPS−induced oxidative stress in brain tissue, thereby mitigating LPS−induced neuroinflammation.

### 2.5. OXY Alleviated LPS-Induced Neuroinflammation in Brain

**Figure 7:**
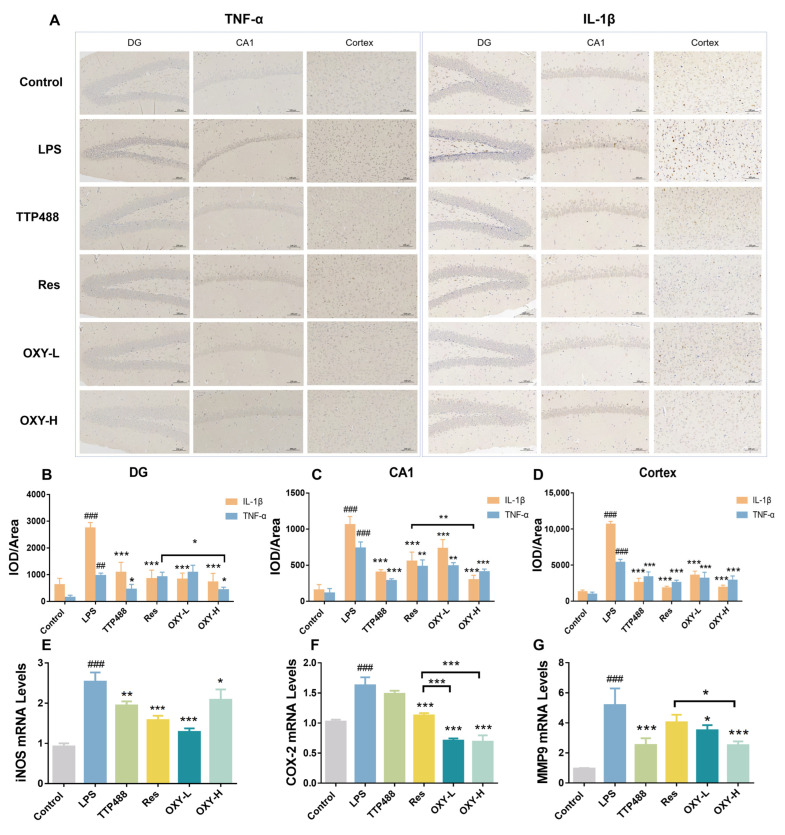
The expression levels of inflammatory factors in the mouse brains. (**A–D**) The expression of TNF−α and IL−1β were measured by IHC; (**E–G**) The mRNA levels of iNOS, COX−2 and MMP9 were measured by RT−PCR. All values are mean ± S.D. (*n* = 3), * *p* < 0.05, ** *p* < 0.01, *** *p* < 0.001, treatment groups versus LPS group, ^##^ *p* < 0.01, ^###^ *p* < 0.001, LPS group versus control group. One−way ANOVA followed by Tukey’ s comparison test.

As shown in Figure 7A–D, the expression of TNF−α in the hippocampus and cortex is higher in the LPS group than in the control group. After treating with OXY, the expression of TNF−α in DG, CA1 and the cortex part all significantly decreased. More importantly, in the DG part, the inhibiting effect of TNF−α in the OXY−H group was more significant than in the Res group. Also, in Figure 7A–D, compared with the control group, the expression of IL−1β in the LPS group in the DG, CA1 and cortex part were significantly increased. After OXY treatment, the expression of IL−1β was attenuated. As well as in the CA1 part, the inhibiting effect of IL−1β in OXY−H group was better than in the Res group.

The mRNA levels of iNOS, COX−2 and MMP9 in mouse brains were quantitatively determined by real−time PCR. As shown in Figure 7E–G, the mRNA levels of iNOS, COX−2 and MMP9 in the LPS group were upregulated. The treatment of resveratrol and OXY could both inhibit the mRNA levels of iNOS, COX−2 and MMP9.

### 2.6. OXY Inhibited the Activation of the PI3K-Akt Signaling Pathway (Network Pharmacology Analysis)

**Figure 8:**
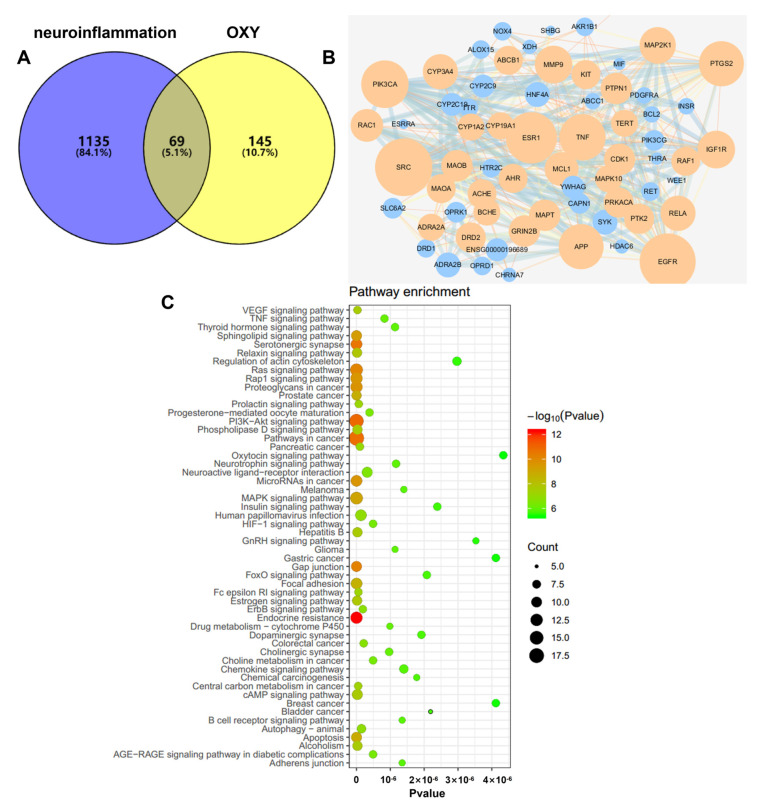
(**A**) Venn diagram: 69 overlapping targets between predicted targets of OXY and neuroinflammation-associated targets. (**B**) PPI network: The PPI network was constructed using Cytoscape and analyzed by using Network Analyzer. Orange and blue colors represent the upregulation and downregulation of the target, respectively. Node size is proportional to the degree of interaction. (**C**) KEGG enrichment analysis of the anti−neuroinflammation targets of OXY.

Data on neuroinflammation−related targets retrieved from Targetnet, UniProt and the Genecards were integrated, resulting in the retrieval of 1204 targets. Then, these targets were compared with the predicted targets of OXY, and 69 common targets were filtered as the key targets for testing the anti−neuroinflammation activity of OXY (Figure 8A).

To explore the possible mechanism of OXY as a treatment against neuroinflammation, 69 targets were imported to the STRING database in order to construct an original protein–protein interaction (PPI) network. The final network was constructed from the tsv file of the PPI data generated in STRING by using Cytoscape (version 3.6.0), which comprised 67 nodes and 349 edges (Figure 8B). The main targets of OXY in regulating neuroinflammation include SRC, EGFR, ESR1, PIK3CA, TNF, PTGS2, etc.

A KEGG pathway enrichment analysis at *p* < 0.01 significance level was performed on the 69 common targets (Figure 8C). Based on the results, we speculated that the mechanism of action of OXY-inhibiting neuroinflammation is possibly through regulating the PI3K−Akt signaling pathway.

## 3. Materials and Methods

### 3.1. Chemicals and Reagents

Oxyresveratrol (98%, Sun Yat-sen University, Guangzhou, China), resveratrol (98%, Kanglun Biotechnology Co., Ltd., Guangzhou, China). Nitric Oxide Kits (Beyotime Biotechnology, Shanghai, China), MTT (Sigma-Aldrich, St, Louis, MO, USA). The BV-2 cell lines (BNCC337749) were purchased from BeNa Culture Collection (Beijing, China), Superoxide dismutase (SOD) and reactive oxygen species (ROS) detection kits were purchased from Jiangsu Meibiao Biotechnology Co., Ltd. (Yancheng, Jiangsu, China). The LPS-PG (Lipopolysaccharide from *P. gingivalis*) was purchased from InvivoGen (Hongkong, China). The COX-2, IL-1β, TNF-α, Arg-1 antibodies (rabbit anti-mouse) and secondary antibody (goat anti-rabbit) were provided by Servicebio Technology Co., Ltd. (Wuhan, China).

### 3.2. Cell Culture

Cell culture reagents were purchased from Gibco (Life Technologies, Gaithersburg, MD, USA) unless otherwise illustrated. The BV-2 cells were maintained in DMEM medium. All of the media were supplemented with 10% (*v*/*v*) heat-inactivated FBS, 100 units/mL penicillin-streptomycin. The cells were incubated at 37 °C in a humidified atmosphere of 5% CO_2_. When the cells reached 80% confluence in culture flasks, trypsin-EDTA was used to detach the cells and the cells were used in experiments or reseeded in the flask.

### 3.3. Cell Viability and Measurement of NO Production

Assessment of cell viability was performed using the MTT assay following the previously published protocol [20]. Briefly, 5 × 10^3^ cells were seeded in 96-well plates and grew for 24 h before treatment with OXY (12.5, 25, 50, 100, 200, 400, 800 μM) for another 24 h. MTT reagent (0.5 mg/mL in DMEM) was added to each well, and cells were incubated at 37 °C, 5% CO_2_ for 4 h. The culture supernatants were discarded, and 200 µL of DMSO was added to each well. Assays were performed in triplicate. The absorbance at 540 nm was measured using a microplate reader (Thermo Fisher Scientific, Waltham, MA, USA). The percentage of surviving cells is calculated according to the equation: viability of cells (%) = (A_sample_ − A_blank_)/(A_control_ − A_blank_) × 100. A_sample_, A_blank_ and A_control_ represent UV absorption at 540 nm for cells treated by therapeutic agents, culture medium or vehicle, respectively.

Assessment of OXY attenuating LPS-induced inflammation was performed using the Nitric Oxide Kits Assay. Briefly, 10^5^ cells were seeded in 96-well plates and grew for 24 h before treatment with OXY (12.5, 25, 50, 100, 200 μM) + 100 ng/mL LPS for another 24 h. Then, 50 μL Griess A and 50 μL Griess B were added to each well for 5 min. At last, the absorbance at 540 nm was measured.

### 3.4. Immunofluorescence Staining

BV-2 cells were seeded at the density of 2 × 10^4^ per cover glass fixed in 6-well plates and incubated overnight. Cells were pre-treated with 50 μM OXY for 2 h prior to treatment with 0.5 μg/mL LPS. After 24 h exposure, the culture medium was removed. The cells were washed with PBS and then fixed with 4% paraformaldehyde. Cells were then blocked in 0.1% Triton X 100 and 3% BSA for 1 h at room temperature. Primary antibodies COX-2, IL-1β, TNF-α and Arg-1 were incubated overnight at 4 °C. The cells were washed and treated with secondary antibody at a 1:500 dilution for 50 min. The cells were counterstained with DAPI. Subsequently, the cells were washed and imaged using fluorescence microscope (NIKON ECLIPSE C1).

### 3.5. Animal Study

Male C57BL/6 mice weighing 18–20 g were purchased from Guangdong Medical Laboratory Animal Center, China. All experimental studies were approved by the Animal Ethics Committee of South China University of Technology. Mice were fed in the animal facility with free food and water, in a 12 h/12 h light/dark cycle, in constant temperature (22 ± 2 °C) and relative humidity (55 ± 9%).

For experiments, mice were randomly assigned to 6 groups (n = 6/group): control group and LPS group, administered daily with 0.1 mL of 0.9% saline solution per animal by oral gavage (i.g.); LPS + TTP488 group, administered daily with TTP488 (5 mg/kg in 5% CMC-Na solution), LPS + Res group, administered daily with resveratrol (100 mg/kg in saline solution), LPS + OXY-L group, administered daily with low dose of OXY (50 mg/kg in saline solution) and LPS + OXY-H group, administered daily with a high dose of OXY (100 mg/kg in saline solution). All treatments were carried out for 14 consecutive days. Meanwhile, modeling operation (intraperitoneal injection of LPS) began on Day 8 of the experiment [21]. Each animal in the control group was injected daily with phosphate-buffered saline (PBS), while animals in the LPS groups were injected daily with 250 μg/kg LPS for 7 days. The experimental process is shown in Figure 1B.

The Morris water maze experiment was started on Day 14 of the experiment [22]. It aims to examine the memory and cognitive functions of mice by training mice to learn to find the rescue platforms hidden underwater. The Morris water maze experiment includes a directional navigation experiment and space exploration experiment. The mice were trained 5 days before the water maze experiment, and the directional navigation experiment was conducted on Day 6. On Day 7, the life-saving platform was removed, and the space exploration experiment was carried out.

After the Morris water maze experiment, animals were sacrificed under spinal dislocation and their blood and brains were immediately removed for further analysis.

### 3.6. Histopathology and Immunohistochemistry

The brains were immediately fixed in 4% paraformaldehyde at 4 °C for 24 h and then in 50% ethanol before processing for histological studies. Serial sections (4 μm thickness; 1 section per slide) were performed for histopathological analysis. Sections were subjected to Nissl staining and were examined using a light microscope (Nikon Eclipse E100, Tokyo, Japan). Fields were selected following a systematic random sampling scheme, and the number of neurons was measured using Image pro plus. For IHC studies, after deparaffinization and dehydration, sections were washed with 3% H_2_O_2_ for 10 min at room temperature, and non-specific binding of immunoglobulins was blocked with 2% normal goat serum (diluted 1:10 in PBS) for 30 min at room temperature. Sections were boiled for 30 min in critic acid (pH 6.0) for antigen retrieval. After blocking, sections were incubated overnight at 4 °C with primary antibodies, as follows: IL-1β, TNF-α. Then sections were washed and treated with secondary antibody at a 1:500 dilution for 30 min. Images were acquired using a light microscope (Nikon Eclipse E100, Tokyo, Japan).

### 3.7. Enzyme-Linked Immunosorbent Assay (ELISA)

Brain tissue levels of reactive oxygen species (ROS) and superoxide dismutase (SOD) were measured by the ELISA kits to investigate oxidative stress in brain tissue in mice. All protocols were performed by following the manufacturer’s instructions.

### 3.8. Real-Time PCR Analysis

Brain tissues were homogenized in liquid nitrogen, and total RNA was extracted using an easy-RED™ (iNtRON, Seongnam, Republic of Korea), and it was reverse transcribed into cDNA using a PrimeScript™ 1st strand cDNA Synthesis Kit (TaKaRa, Tokyo, Japan) according to the manufacturer’s protocol. The primer information is shown in Table 1.

### 3.9. Network Pharmacology

Targets screening: The targets of oxyresveratrol were predicted by the Drugbank database “https://www.drugbank.ca/ (accessed on 28 March 2022)”, the Targetnet database “http://targetnet.scbdd.com/calcnet/index/ (accessed on 28 March 2022)”, and the published literature. *Homo sapiens* were selected as the target organism. Data on the neuroinflammation-associated protein targets were obtained from the Swisstarget Prediction database “http://swisstargetprediction.ch (accessed on 28 March 2022)”, the Targetnet database “http://targetnet.scbdd.com/calcnet/index/ (accessed on 28 March 2022)”, the UniProt database “https://www.uniprot.org (accessed on 28 March 2022)”, the Genecards database “https://www.genecards.org/ (accessed on 28 March 2022)”, the KEGG database “http://www.kegg.jp/ (accessed on 28 March 2022)”. A gene library of the anti-neuroinflammation targets of oxyresveratrol was established by comparing and analyzing the targets common between the neuroinflammation-associated targets and the predicted oxyresveratrol targets.

Network construction and pathway enrichment: Venny “https://bioinfogp.cnb.csic.es/tools/venny/ (accessed on 31 March 2022)” analyzed the collected neuroinflammation target library and the oxyresveratrol target library to obtain the oxyresveratrol-neuroinflammation target intersection. The STRING database “https://string-db.org/ (accessed on 31 March 2022)” was used to establish the relationship between the targets, and Cytoscape 3.7.2 was used to analyze the network characteristics of the oxidized resveratrol target association network. Lastly, the KEGG database was used to analyze the cross targets and obtain the mechanism pathway enrichment diagram.

### 3.10. Statistical Analysis

All quantitative data are expressed as mean ± S.D. and all comparisons were made with 6 animals per group using one-way ANOVA followed by Tukey’s comparison test (GraphPad Software, version 6.02 for windows, San Diego, CA, USA); *p* < 0.05 was considered statistically significant.

## 4. Discussion

The present study demonstrated that administering OXY effectively improves cognitive impairments and episodic-like memory by transforming BV-2 from M1 proinflammatory phenotype to M2 anti-inflammatory phenotype, attenuating LPS-induced neuroinflammation and neuronal damage in the hippocampus and cortex. The protective effect of OXY on neuroinflammation is supposed to be through inhibiting the phosphorylation process of the PI3K-Akt pathway to downregulate the expression of inflammatory factors, TNF-α, IL-1β, iNOS, COX-2 and MMP9.

The deterioration of cognitive and memory is partially attributed to an accumulation of molecules resulting from oxidative stress in the brain, such as reactive oxygen species (ROS), and molecules derived from inflammatory states. These molecules trigger the production of various cytokines and interleukins (IL) that collectively induce neuroinflammation [23,24], a phenomenon referred to as inflammation when it occurs during the aging process [25]. Neuroinflammation results in brain damage that leads to cognitive and memory deterioration due to the lack of an adequate physiological response to counteract it due to nerve wear and tear caused over years [26]. The hippocampus, one of the brain regions associated with memory [27], is also involved in spatial navigation and motor sequence memory, completely affecting movement.

Neuroinflammation, a defense mechanism aimed to protect the CNS, has been suggested to contribute to neurodegenerative diseases and cognitive dysfunction. Microglia, the main executor of neuroinflammation, are considered as the resident macrophages in the brain, which play multiple roles against potentially dangerous agents [28]. Recently, a lots of evidence has reported that LPS activate microglia both in vivo and in vitro [29,30,31]. The activation of microglia cells by LPS produces a massive amount of pro-inflammatory cytokines, ROS and nitric oxide (NO), which may further cause neuronal damage. Intraperitoneal injection of LPS to murine has been established as a model of neuroinflammation, since this route of administration provokes a systemic inflammation response, which can reach the CNS through the blood circulation [19,29]. Thus, LPS is widely used to induce neuroinflammation [32,33,34]. In this research, the neuroinflammation mouse model was established by intraperitoneal injection of LPS, presenting a typical phenotype of neuroinflammation. Compared with the dosage of 500 or 750 μg/kg/day used in the research, 250 μg/kg/day injection dose was found to produce significant phenotypes in mice. Excessive expression of inflammation factors ROS, SOD, TNF-α, IL-1β, iNOS, COX-2 and MMP9 was speculated as the inducer of neuronal injury contributing to behavioral deficits. In both in vitro and in vivo neuroinflammation models, OXY exhibited a great potential to attenuate LPS-induced pathologic changes.

In this study, resveratrol and TTP488 were both introduced as positive control drugs. Resveratrol inhibits microglial activation and regulates neuroinflammation to exert neuroprotective effects in Alzheimer’s disease and Parkinson’s disease [35,36,37]. OXY (PubChem CID: 5281717) is one of the natural analogues of resveratrol (PubChem CID: 445154), both of which are plant-derived polyphenols. To our knowledge, the anti-neuroinflammatory effect of OXY in vivo has not been elucidated yet, though a recent study reported OXY to inhibit the IL-1β-induced activation of HMC3 human microglia [38]. Here we established a neuroinflammation mouse model and confirmed that OXY can improve cognitive impairments and episodic-like memory through modulating neuroinflammation and PI3K-Akt signaling pathway for the first time. It is found that OXY treatment can reduce the apoptosis of hippocampus neurons and inhibit the oxidative stress factors and inflammatory factors in brain tissue. It also exhibited similar therapeutic effects as its analogue resveratrol at the same dose (100 mg/kg/day) in animal behavioral tests, but a superior effect in suppressing TNF-α, IL-1β, COX-2 and MMP9 release in the CA1 region and the cortex.

The research also suggested that OXY inhibited the polarization of BV-2 microglia towards M1 and promoted the polarization of microglia towards M2 It has been well known that microglial functional plasticity is stimuli dependent. M1 activation of microglia leads to the release of proinflammatory factors, while M2 activation turns out to be anti-inflammatory and neuroprotective [39]. The two phenotypes of microglia are mainly characterized by the expression of typical markers. In parallel with the previous study implicating the anti-inflammatory role of resveratrol [40], we observed in BV-2 microglia the upregulation of M1 phenotype makers IL-1β and TNF-α, and downregulation of M2 phenotype marker Arg-1 by OXY. Furthermore, OXY was found to alleviate LPS-induced proinflammatory cytokines and factors in the mouse brain, and to improve behavioral performance in cognition-impaired model mice. As suggested by the previous research [41], the beneficial effects of OXY in vitro and in vivo might be attributed to its property of microglia polarization modulation.

When there are stimulatory factors such as LPS, IFN-γ, etc., TLR4 receptors transmit external stimuli such as LPS into the cells, activate PI3K-Akt, MAPK and other signaling pathways, and IκB is activated and finally degraded by phosphorylation and ubiquitination; NF-κB is released, phosphorylated, and transferred to the nucleus to induce the expression of inflammatory mediators; it eventually leads to the secretion of pro-inflammatory factors (IL-1β, TNF-α, iNOS, COX-2 and MMP9). Increased expression of iNOS and COX-2 are the symbols of activated microglia. MMP9 has been shown to be involved in the occurrence and development of inflammation, and the application of MMP9 inhibitors can reduce the inflammatory response in the brain. Through network pharmacological analysis, the target of OXY-inhibiting neuroinflammation was mainly concentrated on the PI3K-Akt signaling pathway. In the future, we will verify the signaling pathway in experiments.

## 5. Conclusions

In conclusion, this study provides the first evidence that OXY possesses great potential to modulate microglia polarization and improve cognitive impairments and episodic-like memory through modulating neuroinflammation and PI3K-Akt signaling pathway in mice. Therefore, it strongly supports that OXY is neuroprotective against lesions caused by neuroinflammation in vivo and vitro and could be serve as a promising candidate to develop anti-neuroinflammation agents for inflammation-related CNS diseases.

## Figures and Tables

**Table 1:** Primer information.

Gene		Sequence (5′-3′)
COX-2	Forward:	ATAGACGAAATCAACAACCCCG
	Reverse:	GGATTGGAAGTTCTATTGGCAG
iNOS	Forward:	AGCTCGGGTTGAAGTGGTATG
	Reverse:	CACAGCCACATTGATCTCCG
MMP9	Forward:	GCTGGCAGAGGCATACTTGTAC
	Reverse:	GGTGTTCGAATGGCCTTTAGTG

## Data Availability

The data of this study are available from the corresponding authors upon request.

## Abbreviations

Aβ	amyloid β
AD	Alzheimer disease
BBB	Blood Brain Barrier
CNS	central nervous system
COX-2	cyclooxygenase-2
IL-1β	interleukin 1 beta
iNOS	inducible nitric oxide synthase
LPS	lipopolysaccharide
MMP9	matrix metalloproteinase 9
OXY	oxyresveratrol
PVDF	polyvinylidene fluoride
Res	resveratrol
ROS	reactive oxygen species
SDS	sodium dodecyl sulfate
SOD	superoxide dismutase
TNF-α	tumor necrosis factor alpha
